# Prevalence and risk factors for methicillin-resistant *Staphylococcus aureus* (MRSA) carriage on admission to different hospital sectors in two European countries

**DOI:** 10.1186/1753-6561-5-S6-O81

**Published:** 2011-06-29

**Authors:** A Lee, Y Carmeli, A Chalfine, L Derde, S Malhotra-Kumar, JA Martínez, J Salomon, A Torres, J Vidal, S Harbarth, the MOSAR WP3, 4 and 5 Study Teams

**Affiliations:** 1 University of Geneva Hospitals, Geneva, Switzerland; 2 Tel Aviv Sourasky Medical Center, Tel Aviv, Israel; 3 Groupe Hospitalier Paris Saint-Joseph, Paris, France; 4 Julius Center for Health Sciences and Primary Care, Utrecht, Netherlands; 5 University of Antwerp, Antwerp, Belgium; 6 Hospital Clinic de Barcelona, Barcelona, Spain; 7 Berck Maritime Hospital, Berck, France; 8 Guttmann Institute, Badalona, Spain

## Introduction / objectives

Knowledge of local MRSA epidemiology at both facility and ward level helps target control measures. This study aimed to determine the prevalence of and risk factors for MRSA colonisation on admission to different healthcare sectors in two European countries.

## Methods

Four centres in Spain and France enrolled in 3 intervention trials of MRSA control performed universal MRSA screening on admission to intensive care units (ICUs), surgical wards and rehabilitation units from August 2008 to March 2010. Demographic and comorbidity data were collected. Univariate and multivariate logistic regression analyses were used to identify risk factors for unknown MRSA carriage on admission.

## Results

Overall, 1780 previously unknown MRSA carriers were screened, 403 (23%) in ICUs, 1099 (62%) in surgical wards and 278 (16%) in rehabilitation wards. The prevalence of unknown MRSA carriage on admission was 2.7% in ICUs, 3.6% in surgical wards and 7.9% in rehabilitation units. No independent risk factors for MRSA carriage on admission to the ICUs were found. Risk factors for surgical wards were age, wounds, nursing home residency and tracheostomy. Rehabilitation unit risk factors were renal failure, diabetic foot, recent antibiotic use and neuro-rehabilitation.

## Conclusion

Prevalence and risk profiles for MRSA carriage on admission to different healthcare sectors varied widely, emphasising the importance of local surveillance data to enable adaptation of MRSA control policies at the ward level.

## Disclosure of interest

None declared.

